# Access to artemisinin-based anti-malarial treatment and its related factors in rural Tanzania

**DOI:** 10.1186/1475-2875-12-155

**Published:** 2013-05-07

**Authors:** Rashid A Khatib, Majige Selemani, Gumi A Mrisho, Irene M Masanja, Mbaraka Amuri, Mustafa H Njozi, Dan Kajungu, Irene Kuepfer, Salim M Abdulla, Don de Savigny

**Affiliations:** 1 Ifakara Health Institute, Dar es Salaam, Tanzania; 2 Swiss Tropical and Public Health Institute, Basel 4002, Switzerland; 3 University of Basel, Basel 4001, Switzerland; 4 INDEPTH Network Effectiveness and Safety Studies of Antimalarial in Africa (INESS), Accra, Ghana

## Abstract

**Background:**

Artemisinin-based combination treatment (ACT) has been widely adopted as one of the main malaria control strategies. However, its promise to save thousands of lives in sub-Saharan Africa depends on how effective the use of ACT is within the routine health system. The INESS platform evaluated effective coverage of ACT in several African countries. Timely access within 24 hours to an authorized ACT outlet is one of the determinants of effective coverage and was assessed for artemether-lumefantrine (Alu), in two district health systems in rural Tanzania.

**Methods:**

From October 2009 to June 2011we conducted continuous rolling household surveys in the Kilombero-Ulanga and the Rufiji Health and Demographic Surveillance Sites (HDSS). Surveys were linked to the routine HDSS update rounds. Members of randomly pre-selected households that had experienced a fever episode in the previous two weeks were eligible for a structured interview. Data on individual treatment seeking, access to treatment, timing, source of treatment and household costs per episode were collected. Data are presented on timely access from a total of 2,112 interviews in relation to demographics, seasonality, and socio economic status.

**Results:**

In Kilombero-Ulanga, 41.8% (CI: 36.6–45.1) and in Rufiji 36.8% (33.7–40.1) of fever cases had access to an authorized ACT provider within 24 hours of fever onset. In neither of the HDSS site was age, sex, socio-economic status or seasonality of malaria found to be significantly correlated with timely access.

**Conclusion:**

Timely access to authorized ACT providers is below 50% despite interventions intended to improve access such as social marketing and accreditation of private dispensing outlets. To improve prompt diagnosis and treatment, access remains a major bottle neck and new more innovative interventions are needed to raise effective coverage of malaria treatment in Tanzania.

## Background

Following high investment in the fight against malaria, very important progress has been made for its control across Africa, including Tanzania [1-8]. However, the disease still remains an important public health problem in the country and accounts for nearly one third of the country’s disease and mortality burden [9]. Children under five years of age and pregnant women are at highest risk of the disease and its subsequent adverse outcomes. Even though, as the overall burden is decreasing, the burden is shifting towards older children and young adults [10].

Prompt recognition and timely treatment with efficacious drugs remains a primary control strategy for malaria [11]. Artemisinin-based combination therapy (ACT) has been recommended as the drug of choice for the treatment of uncomplicated malaria and have widely been adopted as first line treatment in sub-Saharan Africa [12]. Early diagnosis and treatment are crucial in achieving the drug’s intended benefits. Proliferating malaria parasites which are responsible for life-threatening malaria complications and fostering transmission can be rapidly and effectively controlled if effective treatments are taken within 24h of onset of clinical signs and symptoms [13]. Several studies have demonstrated an association between delayed treatment and appreciable level of malaria mortality [14-16]. Therefore, timely access to an authorized outlet of ACT within 24 h is one determinant of effective coverage. To reduce the malaria mortality by 50%, the Roll Back Malaria partnership has set a target of 80% for timely access [17]. The Malaria Eradication (malERA) Consultative Group on Health Systems and Operational Research has introduced the systems effectiveness decay framework [18] that has defined effective coverage in terms of conditional probabilities of population access, accurate targeting, provider compliant delivery and patient adherence. While malaria elimination and eradication are on top of the global agenda, correct and effective treatment is one of the main drivers towards the prerequisite of malaria control.

Tanzania was one of the first groups of countries to implement health systems studies using the systems effectiveness framework to evaluate ACT in the routine health system. Separate modules were used for assessing each dimension of the framework (access, diagnostic targeting, provider compliance and patient adherence). This paper presents results on timely access of fever patients to authorized ACT treatment outlets. Further work on other dimensions will be published separately.

Since 2007, artemether-lumefantrine is the first line treatment for uncomplicated malaria in Tanzania [19,20]. The drug is dispensed in officially-authorized treatment outlets which include government and non-governmental hospitals, health centres, dispensaries, pharmacies and private but accredited drug dispensing outlets (ADDOs). ADDOs are small privately operated retail outlets in rural and peri-urban communities in Tanzania. These outlets whose staff are trained by the government are licensed to sell a set list of essential medicines, including selected prescription drugs, such as ACT. It is a strategy that was intended to expand access to treatments for some common illnesses such as malaria. It was rolled out throughout the country in phased form since 2007. This study compares areas with and without ADDOs.

## Methods

### Study area and population

Data for this study were collected within the framework of the Rufiji and Kilombero-Ulanga HDSS sites. Both sites are located in the Greater Rufiji River Basin in southern Tanzania. The sites are primarily rural with majority of the population relying on subsistence farming or fishing. Both sites are characterized by heavy rains from March to May.

### Rufiji

Villages constituting Rufiji HDSS site are situated in Rufiji District, Coast Region and cover a total of 16,685 households and 84,095 residents in 38 villages. The villagers belong to varied ethnic groups, the biggest being Ndengereko, Matumbi, Nyagatwa and Ngindo. Islam is the dominant religion in the area.

### Kilombero-Ulanga

Kilombero-Ulanga HDSS includes 28,000 households with a total population of 120,000 in 25 villages in the districts of Kilombero and Ulanga. The main ethnic groups are Ndamba, Pogoro and Hehe. The population is equally divided between Christians and Muslims.

During the time of our survey there were 24 health facilities and a limited number of ADDOs in Rufiji HDSS and there were 14 health facilities and 44 ADDOs in KU.

In Rufiji, the population level of malaria parasitaemia is 15% and in KU 13% [7]. In both sites, malaria was historically intense [21-27]. Important progress has been made in its control, and population parasitaemia levels have fallen from 26% in 2001 to 15% in 2006 in Rufiji and from 18% to 13% over the same period in KU [7], yet the disease still remains one of the key public health problems. However, malaria transmission in the districts is still endemic, most common from June to September, the period after the long rain season.

Fever has been used as the defining indicator of malaria and every fever has long been treated as malaria in the area as it for the rest of the country. Health facility based studies have of late demonstrated a significant decline of malaria parasitaemia among febrile patients in health facilities. Only 33% of fever patients were diagnosed with malaria using malaria Rapid Diagnostic Tests (RDT) from this district’s health facilities [28]. Existing observations suggest malaria parasitaemia among patients with fever complaints in health facilities was 20% in this site [28]. However, intermittent RDT stock-outs and continuing treatment of malaria without parasitological confirmation mean that ACT continues be used in the majority of fever cases.

### Sample size and sample size calculation

Sample size was calculated based on the assumption of 50% of the fever cases seek and gain access, an annual fever prevalence of 6%, a design effect of 1.5 to account for clustering within households, and a dropout rate of 10%. To estimate the proportion of patients that gain access to an ACT provider within 24/48 hours after onset of fever with a precision ±5%, a minimum of 1,152 interviews needed to be conducted in each site.

### Procedures

In each HDSS household an interview was conducted as part of the routine HDSS update rounds. From October 2009 to June 2011 a total of 4,648 and 4,226 households were randomly pre-selected from the Rufiji and KU household registers respectively. In each HDSS the survey was conducted continuously and longitudinally, sampling covering pre-selected households without replacement over one calendar year to capture periods of higher and lower malaria transmission. During HDSS site routine household visits, each member was asked if they had fever or malaria in the preceding 14 days. If yes, they were eligible for the in-depth interview after written informed consent was obtained. For children under 12 years approval was obtained from their parents or guardians. The interview was conducted at household level. Data on individual treatment-seeking, access to treatment, services sought, drugs provided, and associated household cost details for all costs associated with the episode were captured. Field workers carried photo albums with views of all possible malaria treatment packaging in order to identify drugs provided. The socio-economic status of each household was obtained from the asset surveys from the HDSS.

### Ethical approval

Ethics approval for the study was granted by the institutional review boards of Ifakara Health Institute (IHI), and by the National Tanzanian Medical Research Co-ordinating Committee of the National Institute for Medical Research (NIMR).

### Data analysis

Data were entered using EpiData (Odense, Denmark) and transferred into STATA version 10 software (Stata Corp, College Station, TX) for merging, cleaning and performing analyses. The main outcome measure was the proportion of fever patients that sought treatment from an authorized ACT provider within 24 hours of fever onset. Secondary outcomes evaluated were: a) the proportion of fever patients that sought treatment from an authorized ACT provider within forty eight hours of the onset of fever; and b) the proportion of malaria patients that sought treatment from an authorized ACT provider after forty eight hours of the onset of fever. Comparison of these outcomes within and between the two study sites was made using Chi-square test. Logistic regression was used to assess the importance of the selected determinants explored in the study. The Concentration Index formula by Kakwani *et al*. [29] was used to generate concentration indices from the study to identify existence of socio-economic inequality in 24 hour access to ACT outlets.

## Results

### Description of the sample

A total of 1,041 and 1,103 interviews were conducted in Rufiji and Kilombero-Ulanga HDSS sites, respectively. The sample composition was almost equally divided between sexes and people of different socio-economic categories (Table 1). Over two thirds of study participants were over five years old. As expected, the majority of fever/malaria episodes were identified during high malaria transmission season from both HDSS sites. The study sites are characterized by endemic malaria transmission with high seasonal variation, and as shown in Table 1, over 60% of interviews from both Rufiji and Kilombero-Ulanga were conducted during high malaria transmission season. Health facility visits on occurrence of fever/malaria were most common in both sites, ranging from 47% in KU to 48% in Rufiji. Visits to unauthorized malaria treatment outlets were more common in Rufiji (40.5%) than in Kilombero-Ulanga (14.7%). These unauthorized outlets were predominantly normal shops and street kiosks. There were patients who reported treatment visits to both health facilities and ADDOs; 4.2% were observed in Rufiji and 5% in Kilombero-Ulanga. Equally important, not more than 35% of patients from Rufiji and around 19% from Kilombero-Ulanga reported being diagnosed using either RDTs or microscopy.

**Table 1:** Charateristics of study population in Rufiji and Kilombero-Ulanga (KU) HDSS sites, Tanzania

	**Rufiji**	**KU**
Number of peopple identified with recent fever	1041 (100%)	1103 (100%)
Number of people with recent fever who completed access interviews	1024 (98.4%)	1088 (98.6%)
Number of people with recent fever with incomplete access interviews	4 (0.4%)	15 (1.4%)
Number of people with recent fever with no access interviews	13 (1.3%)	0
**Of those with complete interiviews**		
**Sex**		
Males	442 (43.2%)	466 (42.8%)
Females	582 (56.8%)	622 (57.2%)
**Age groups**		
Under-five children	328 (32%)	300 (27.6%)
Five years and above	696 (68%)	788 (72.4%)
**Socio-economic status by asset index**		
Poorest	194 (19%)	176 (16.2%)
Less poor	201 (19.6%)	244 (22.4%)
Middle	192 (18.8%)	196 (18%)
More rich	229 (22.4%)	204 (18.8%)
Least poor	208 (20.3%)	268 (24.6%)
**Complete interviews conducted during:**		
High malaria transmission season	618 (60.4%)	736 (67.7%)
Low malaria transmission season	406 (39.7%)	352 (32.4%)
**Use of different malaria treatment providers**		
Health facilities	498 (48.6%)	511 (47%)
Accreditted Drugs Dispensing Outlets (ADDOS)	97 (9.5%)	395 (36.3%)
Other authorized treatment providers	14 (1.4%)	22 (2%)
Unauthorized providers	415 (40.5%)	160 (14.7%)
Both health facilities and ADDOs	43 (4.2%)	54 (5%)
Having a blood diagnosed using mRDT or microscopy	332 (32.4%)	199 (18.3%)

As shown in Table 2, timely access to an authorized ACT provider was 37% (CI:33.7–40.1) in Rufiji DHSS and 42% (36.6–45.1) in Kilombero-Ulanga. Statistically significant difference was observed in access within 48 hours between the two sites; higher from Kilombero-Ulanga 70% (CI:67.3–73.3) compared to Rufiji 53% (CI:49.9–56.7). Proportion of patients with access after 48 hours was small and there was no statistically significant difference between the sites. The combined access within 48 hours for the two study sites (calculated from the data but not shown in the figure) was 62%.

**Table 2:** Fever/malaria patients' access to authorized malaria treatment providers in Rufiji and KU

	**Rufiji (N=1024)**		**KU (N=1088)**	
	**n (%)**	**95% CI**	**n (%)**	**95% CI**
Access to authorized providers within 24 h	377(36.8)	33.7-40.1	455(41.8)	38.6-45.1
Access to authorized providers within 48 h	546(53.3)	49.9-56.7	766(70.4)	67.3-73.3
Access to authorized providers after 48 h	205(20)	17.5-22.8	200(18.4)	16.0-21.0

Distribution of access to ACT providers within 24 hours by malaria transmission season, sex, age groups and socio-economic status is highlighted in Table 3. No difference was observed between males and females from both sites. Under-fives from both sites benefited better than the rest of the population, however, this variation did not reach statistical significance in either study area. In terms of socio-economic status, no evidence of inequality between quintiles and access to malaria treatment providers within 24 hours was proven from both sites. Concentration Index measured from Rufiji was 1.78 (CI:–0.01–3.57). The index from KU was 1.49 (CI: 0.01–2.99). Timely access was shown to be the same for patients between high and low malaria transmission seasons from both sites.

**Table 3:** Distribution of access within 24 h by sex, age, SES and seasonality in Rufiji and KU

	**Rufiji (N=1024)**		**KU (N=1088)**	
	**n (%)**	**95% CI**	**n (%)**	**95% CI**
**Distribution by seasonality**				
High malaria transmission season	235/618(38.0)	34.0-42.3	323/736(43.9)	39.9-47.9
Low malaria transmission season	142/406(35.0)	30.1-40.1	132/352(37.5)	32.3-43.0
Males	167/442(37.8)	33.1-42.7	196/466(42.1	37.4-46.9
Females	210/582(36.1)	32.1-40.2	259/622(41.6)	37.5-45.9
**Distribution by age groups**				
Under-five children	134/328(40.9)	35.5-46.4	139/300(46.3)	40.4-52.3
Five years and above	243/696(34.9)	31.2-38.9	316/788(40.1)	36.4-43.9
**Distribution by socio-economic status**				
Poorest	64/194(33.0)	26.5-40.2	66/176(37.5)	28.7-47.2
Less poor	73/201(36.3)	29.1-44.2	88/244(36.1)	30.2-42.4
Middle	63/192(32.8)	26.5-39.9	97/196(49.5)	42.1-56.9
More rich	79/229(34.5)	28.1-41.5	88/204(43.1)	36.0-50.6
Least poor	98/208(47.1)	40.0-54.4	116/268(43.3)	37.1-49.7
Concentration Index	1.78	-0.01-3.57	1.49	-0.01-2.99

Table 4 summarizes the results in predictors for timely access. Factors that were considered in the model were sex, age group, type of treatment providers, wealth quintile, sex of caretakers and seasonality in malaria transmission. The model was adjusted to account for any potential confounders. There were no statistically significant differences by sex of patient, age groups, socio-economic status, sex of caretaker or malaria transmission season from both study sites. There was 38% and 34% more likelihood in seeking ACT within 24 hours from ADDOs health facilities in Rufiji and KU respectively but differences were not statistically significant. No difference was observed in both sites for access within 24 hours between health facilities and other providers. Overall, having access within 24 hours from unauthorized providers was less likely compared to health facilities in both sites.

**Table 4:** Predictors of patients' access to authorized malaria treatment providers within 24 hours in Rufiji and KU

**Variable**	**Rufiji**		**Kilombero-Ulanga**	
	**n/N(%)**	**Adjusted odds ratio (95%CI)**	**n/N(%)**	**Adjusted odds ratio (95%CI)**
Male	167/442(37.8)	reference	196/466(42.1)	reference
Female	210/582(36.1)	0.991(0.7-1.3)	259/622(41.6)	1.063(0.8-1.4)
<5 years	134/328(40.9)	reference	139/300(46.3)	reference
≥5 years	243/696(34.9)	1.1(0.7-1.5)	316/788(40.1)	0.739(0.5-1.1)
Health facilities	251/550(45.6)	reference	216/495(43.6)	reference
ADDO	48/91(52.8)	1.383(0.9-2.2)	206/409(50.4)	1.343(1.0-1.8)
Other private	31/50(62.0)	1.886(1.0-3.6)	28/55(59.9)	1.510(0.8-2.7)
Non authorized providers	47/333(14.1)	0.230(0.2-0.5)	5/129(3.9)	0.090(0.3-0.2)
Poorest	64/194(33.0)	reference	66/176(37.5)	reference
Less poor	73/201(36.3)	1.24(0.8-2.0)	88/244(36.1)	0.831(0.5-1.4)
Middle	63/192(32.8)	0.92(0.6-1.4)	97/196(49.5)	1.352(0.8-2.3)
More rich	79/229(34.5)	0.987(0.6-1.5)	88/204(43.1)	1.041(0.6-1.7)
Least poor	98/208(47.1)	1.669(1.1-2.6)	116/268(43.2)	1.112(0.7-1.8)
Patients themselves	136/438(31.1)	reference	189/482(39.2)	reference
Male caretaker	43/108(39.8)	1.417(0.9-2.3)	65/140(46.4)	1.066(0.7-1.6)
Female caretaker	198/478(41.4)	1.433(1.0-2.1)	201/466(43.1)	1.093(0.8-1.5)
Low malaria transmission season	142/406(35.0)	reference	132/352(37.5)	reference
High malaria transmission season	235/618(38.0)	1.204(0.0.9-1.6)	323/736(43.9)	1.345(0.1-1.8)

## Discussion

ACT has been recommended as a life-saving intervention for malaria cases especially in sub-Saharan African countries which claims more than 60% of the global malaria morbidity [30,31]. However, full benefits cannot materialize until important health system elements, including patients’ access, are addressed. It is argued that malaria elimination, an essential step towards the long-term goal of eradication, can only be achieved when a high proportion of patients with malaria have access to effective treatment [31]. This is critical for the malaria eradication agenda. As is the case for many other endemic sub-Saharan African countries, malaria is generally on the decline in Tanzania [1-8]. However, a new push is required to sustain the gains and move towards malaria elimination. It is in this way that this bottleneck needs urgent attention if the potential of ACT is to be optimized and this control agenda is to be fulfilled. It is unfortunate for malaria control initiatives that when substantial resources are being invested in the development and procurement of efficacious drugs, these tools do not reach the patients in a timely way that would contribute to malaria reduction commensurate with their demonstrated capacity. ACT is most effective against malaria when acting on parasites in cases not yet severely complicated [13].

This is one of few studies that have demonstrated patients’ access to ACT within the context of system effectiveness framework [25,26] in two neighbouring HDSS sites located in three districts in Tanzania. Although the results are not representative of the country composed of more than 130 districts, they offer an insight on the health system’s performance and an estimate of timely access to ACT. As they are HDSS sites, their populations are investigated more often and several health system interventions were implemented on a research basis than elsewhere in the country [7,18,21,22,27,28]. Hence it is reasonable to estimate that these study results should not be worse than the rest of Tanzania. The country’s latest Tanzania HIV/AIDS and malaria indicator survey (2011-2012) was a nationwide study, the results of which suggest 21% access to malaria treatment within 24h [32]. Observations from this study and those reported from TDHS 2011 fall short of 80% target set by Roll Back Malaria for achieving the potential public health benefits of ACT [33]. They suggest that more than half of people ill with malaria in Tanzania do not have timely access to quality ACT and are exposed to risk of poor outcomes of the disease. These results are comparable to the rest of rural Africa where malaria transmission is endemic where a similar picture of malaria patients’ access to ACT has been reported in Burkina Faso and Kenya [30,34].

Fever patients’ access to ACT has long been investigated in KU [17], following its implementation of the Access Programme from 2004 to 2007 [23], which involved social marketing campaigns that stressed the importance of prompt and effective treatment for malaria. The KU site was also among the first districts in the country having ADDOs. There were 44 ADDOs in the study area at the time of our survey giving an outlet to population ratio of 0.4 per 1,000. Rufiji was characterized by four ADDOs as the study was in progress with an outlets to population ratio of 0.05 per 1,000. Hence, both social marketing and ADDOs should have increased timely access to appropriate treatment in KU compared with Rufiji but we did not detect a significant difference. This could be interpreted that these interventions did not have an impact on fever patients’ timely access to ACT although access after 24 hours was better in KU.

Timely access to ACT previously observed in KU from the ACCESS programme was above 60% [17]. This is better than the rate reported in the present study. This discrepancy would be explained in two ways. One, it would be associated with different dimensions used to report effective coverage between our surveys and study conducted during the ACCESS project. Alba *et al.* presented actual anti-malarial drug taken by patients within 24 h following fever episode as reported by patients or by their caretakers. Patients would take drugs available at home or from a neighbour or friend and this has been reported in several studies [15,31,35,36]. Wider availability of sulphadoxine-pyrimethamine (SP) and older drugs during ACCESS survey was reported from KU [37]. Thirty percent and 16% of patients self-treated at home from Rufiji and Kilombero-Ulanga respectively from our study (data not shown in the results section). In this study, results presented reflect patient or caretaker reported visit made to ACT providers within 24 h on the onset of fever illness. As reported in the preceding sections, results on which this paper is based have been generated from just one access dimension of effective coverage. Other components proposed in the effectiveness framework (targeting accuracy, prescribers’ compliance and patients’ adherence) [18] have been conducted using different designs whose results are published separately [28]. Findings from these papers will demonstrate whether the patients obtained and used the medicines comparable to ACCESS publications. On the other hand, the social marketing campaigns and ADDOs roll out might have improved timely treatment access for fever as demonstrated in surveys that monitored their effects. And there would be marked difference in timely access between the intervention and comparator site. However, it is also possible that these effects were not sustained over time.

The study has shown more than 15 percentage points statistically significant difference in 48 h patient access between KU and Rufiji sites. The larger ACT outlets population ratio associated with roll out of ADDOs in KU compared to Rufiji could be singled out as an important reason for this difference. It would imply that after the introduction of ADDOs patients have become closer to treatment providers and their number making treatment outlets visits has increased but many of them have not yet started seeking care within 24 h. People still keep delaying to seek treatment from appropriate providers. Observations from some other studies conducted in one of these study areas has suggested an association in this delay with initial actions taken at home with anti-pyretic and locally available anti-malarial medicines [38].

A logistic regression model was run to determine whether there was an association in the observed access with any factors usually estimated as risks for low patients’ access to malaria treatment. This form of analysis assumes that risks of exposure to malaria especially in high endemicity areas and chances for treatment-seeking are not normally distributed [39]. The burden is concentrated in the poorest quintile of the population whose composition is dominated by small children, women and those living in remote rural communities. Hence, malaria control interventions can achieve intensive and broader effectiveness if their coverage is equally clustered around these groups highest at malaria transmission in high burden countries [40]. The risks of inherent effectiveness decay on the poor highlighted in the equity effectiveness loop framework would be addressed when this is properly handled [41]. This study has not been able to prove whether this is happening. Women, under-fives and the poorest of the poor were shown to have almost similar access to ACT providers within 24 hours of malaria onset (Table 4). However, the study cannot claim definite lack of difference in timely access to treatment outlets in these communities by age group or gender as it was not powered to test this. Nevertheless, in a study among the very poor in Tanzania based on observations made in the same area, Schellenberg *et al.* demonstrated unequal access to appropriate treatment between the poorest and the least poor in that area [25]. This difference could have been addressed by subsequent implementation of interventions that used pro-poor approaches [19,42], such as rolling out of ADDOs, and it might also have happened because many public health interventions were implemented in the area generating duplicative effect that has extended to every population group. As was stated earlier, Kilombero-Ulanga and Rufiji HDSS sites were a testing ground for many public health intervention studies in Africa. However, the only condition that the study was powered to demonstrate was access within 24 hours according to malaria transmission season.

This study’s exploration of patients’ access to ACT providers was based on an assumption that fever could be a robust indicator of malaria. Fever has actually been an entry point for several studies seeking to understand treatment-seeking practices for malaria including patients’ access to treatment providers. This has been the case because the widespread adoption of Integrated Management of Childhood Illnesses (IMCI) that encouraged treatment of every fever episode as malaria at a time when malaria transmission was so endemic in Africa and the likelihood of being infected with malaria parasites, especially for small children, was so high. Challenges posed by diagnostic technology in existence at the time and the need to treat patients as timely as possible to prevent progression of the disease into severe form was an important argument raised to justify IMCI strategy. However, as malaria transmission decreases across sub-Saharan Africa, it has been demonstrated that the presence of rapid diagnostic tests is making IMCI no longer an attractive strategy for malaria. Fevers are now increasingly associated with other diseases than malaria, including bacterial infections and especially pneumonia [38,43]. Hence, an assumption that all fevers are malaria is becoming increasingly inappropriate.

## Conclusion

This study showed that access to point of delivery of quality ACT, within 24 h and 48 h is significantly less than 45% and 75% respectively. This is well short of national and global targets despite these being relatively high performing, highly studied districts likely to be better than average in Tanzania. This is not a favourable development in malaria control for the country that is still considered to be high burden. Achieving potential impact associated with ACT, which is essential for progress towards existing malaria control goals, requires the intervention’s universal coverage. It was shown that malaria was falling in Tanzania and the country was moving in the right direction. However, as access to ACT is still low, it would imply that malaria decline is slower than it could be if the drug’s potential was optimized through effective coverage.

In this study, there was no important difference between a district having ADDOs plus social marketing to improve 24 h access to ACT outlets compared to one without. The introduction of ADDOs in KU improved treatment outlets population ratio. However, this development did not translate into higher 24 h access to ACT outlets in KU. There were, however, 48 h access difference between KU and Rufiji and would likely be associated with difference in outlets population ratio. Hence, there is a need for better and more innovative strategies to improve timely access to ACT in Tanzania.

## Competing interests

The authors declare that they have no competing interests.

## Authors’ contributions

RAK contributed to the design of the study, coordinated the study, performed the statistical analyses and interpreted the data, and wrote the manuscript in consultation with the other authors. MBS, MHN and DK performed the statistical analyses and contributed to interpretation of the data and to the drafting and editing of the manuscript. IMM, GAM and MA supervised the survey and contributed to the analysis and interpretation of the data and to the drafting and editing of the manuscript. DdS, IK and SMA conceived of the study and oversaw all aspects of the study, including design and execution of the field work, analysis and interpretation of the data, drafting and editing of the manuscript. All authors read and approved the final manuscript.
